# Gabapentin-Induced Adrenal Insufficiency: The Hypothalamic-Pituitary-Adrenal Axis Stress Misresponse and Risk of Infection: A Case Report and Literature Review

**DOI:** 10.3390/ph17091174

**Published:** 2024-09-05

**Authors:** Evmorfia Pechlivanidou, Alexandros Chatzikyriakos, Maria Anna Zisi, Nikolaos Paraskevopoulos, Semeli Kaltsa, Orestis K. Konstantas, Panteleimon Zogakis, Aikaterini Catsouli, Nick Sekouris, Rodanthi E. Margariti

**Affiliations:** 1 1st Department of Orthopaedics, P. & A. Kyriakou Children’s Hospital, 115 27 Athens, Greece; 2 Department of Hygiene, Epidemiology and Medical Statistics, Medical School, National and Kapodistrian University of Athens, 157 72 Athens, Greece; 3 Medical School, National and Kapodistrian University of Athens, 157 72 Athens, Greece

**Keywords:** gabapentin, adrenal insufficiency, infections, HPA axis, stress responses, *Staphylococcus xylosus*

## Abstract

This literature review, in light of the presented case report, explores the complex interplay between gabapentin (GBP), a gamma-aminobutyric acid (GABA) analog, and the hypothalamic–pituitary–adrenal (HPA) axis in patients undergoing major surgical procedures. It specifically investigates the potential impact of GBP on cortisol levels, stress responses, and infection risk, illustrated by a detailed clinical case. This review combines a comprehensive literature search with a case report of a 17-year-old male with osteosarcoma who experienced transient adrenal insufficiency and infections while receiving GBP. The case is analyzed in the context of the existing literature on GBP and the HPA axis. The findings highlight the intricate relationship between GBP use, adrenal insufficiency, and infection susceptibility. It underscores the need for further research and clinical vigilance when prescribing GBP to patients with underlying medical conditions, particularly in the context of major surgical procedures. The review underscores the need for further research and clinical vigilance when prescribing GBP, particularly in perioperative settings. In conclusion, GBP’s effects on the HPA axis and immune responses are complex and multifaceted. Clinicians should exercise caution when prescribing GBP, especially for patients with underlying conditions undergoing major surgery. Further research is needed to elucidate the mechanisms of GBP’s influence on cortisol levels and stress responses.

## 1. Introduction

Gabapentin (GBP) is a pharmaceutical compound that functions as a gamma-aminobutyric acid (GABA) analog. The FDA has approved it for treating partial seizures and postherpetic neuralgias [1,2]. However, its additional properties as an anticonvulsant, antinociceptive, anxiolytic, and neuroprotective agent also lead to its use off-label. Specifically, doctors commonly prescribe it to manage chronic neuropathic pain [1,2]. The pharmacological mechanism of action relies on the rapid penetration of GBP into cerebral cells [3]. GBP modulates the activity of the glutamic acid decarboxylase (GAD) and branched-chain amino acid transaminase enzymes [4,5]. The cellular mechanisms underlying GBP’s action encompass a variety of processes. Its transportation across various membrane barriers is facilitated by a specific amino acid transporter that competes with leucine, isoleucine, valine (BCAAs), and phenylalanine. Additionally, GBP leads to an elevated concentration of GABA in the brain and an increased rate of GABA synthesis. Furthermore, GBP interacts with voltage-sensitive Ca^2+^ and Na^+^ channels, as well as other neurotransmitters like serotonin [4].

The literature extensively elucidates the significant correlation between GABA and stress [6,7,8,9]. Researchers have observed GABAergic connections on corticotropin-releasing hormone (CRH) neurons located inside the paraventricular nucleus of the hypothalamus (PVN). These neurons play a crucial role in regulating the activity of the hypothalamic–pituitary–adrenal (HPA) axis. Consequently, it appears that GABA exerts a significant regulatory influence over the activity of the hypothalamic–pituitary–adrenal (HPA) axis [7]. Additionally, it has been suggested that gamma-aminobutyric acid (GABA) is an important part of stress reactions. It does this by directly controlling the hypothalamic–pituitary–adrenal (HPA) axis and by causing a lot of biochemical adaptability [6]. Recent research has determined that both phasic and tonic GABAergic inhibition regulate the activity of HPA regulators, including CRH and gonadotropin-releasing hormone (GnRH). Synaptic and extrasynaptic GABA-A receptors (GABAARs) mediate this inhibition. The tonic phase appears to exhibit heightened stimulation during stressful conditions, facilitated by the δ-subunit of GABAARs. This is probably because the ε-subunit is in the best place to control communication between the stress and reproductive axes, especially when neurosteroids from stress are present [10].

Two separate studies [11,12] have revealed a correlation between the introduction of GBP and the suppression of the typical stress response that occurs during the perioperative and postoperative periods. The peripheral antinociceptive action of GBP and its activation of supraspinal brain areas involved in pain signal processing are responsible for this suppression. Additionally, studies have found that the initiation of GBP induces drowsiness [11,12,13,14].

This case report details the presence of hypocortisolemia, a disorder defined by abnormally low levels of adrenocorticotropic hormone (ACTH), which require the initiation of replacement therapy. The condition occurred transiently during a period of high stress connected to surgery, in a patient who had previously undergone prolonged GBP administration in order to avoid postoperative pain. Furthermore, a comprehensive literature analysis is conducted to evaluate the primary pathophysiological, pharmacological, and clinical components of this uncommon case.

## 2. Case Report

At the age of 15, a 17-year-old male, standing at 1.80 m and weighing 118 kg, received a diagnosis of left femur osteosarcoma. The patient was free of personal history and was described as a healthy active boy by his parents. He had complained a few weeks ago about pain in the left femur area and due to findings in the plain X-ray, the patient was referred to the Children’s Hospital by the family’s general practitioner and an orthopedic surgeon. The EURAMOS protocol [15] guided his treatment, which involved tumor excision and implantation of a megaprosthesis for limb salvage [16]. However, he later presented to our department for a revision of the megaprosthesis. Two months after the initial megaprosthesis placement, a Staphylococcus hominis infection necessitated this revision.

Before deciding to proceed with the revision, we performed surgical debridement, which involved the removal of biofilm and the culturing of specimens. Staphylococcus hominis was identified two days after collecting the samples. As a result, the megaprosthesis was removed and replaced with an antibiotic-loaded cement spacer. Tranexamic acid was used as a hemostatic agent during the procedure. A month later, a second-look operation with surgical debridement and a spacer change was performed.

During the period in which the spacer was in place, the patient experienced a massive pulmonary embolism. An interventional radiologist treated this condition by performing a thrombectomy and administering a therapeutic dose of low-molecular-weight heparin (LMWH). There was also a suspected deep vein thrombosis of the left common iliac vein. The patient underwent readmission for the implantation of a new prosthesis almost a year after the removal of the first megaprosthesis. Vacuum-assisted closure (VAC) was used to help repair and heal a large, skin-deficient area. This procedure was successful, and the patient spent one day in the Intensive Care Unit (ICU).

Given the patient’s complex medical history, an extensive laboratory investigation was conducted before his transfer to a regular ward. The results showed that the patient had a morning cortisol plasma level of 1.18 μg/dL, which was below the normal range (normal range: 6.20–23 μg/dL). However, all other hormone levels, including thyroid hormones, fell within the normal range.

Promptly following this discovery, the patient began hydrocortisone replacement therapy, starting with a 100 mg bolus, followed by 200 mg over the course of 24 h. The hydrocortisone replacement therapy was then tapered over several days, with cortisol levels closely monitored until they returned to the normal range. Subsequently, during the post-operative period following the placement of the new prosthesis, the patient developed a significant subcutaneous fluid collection. It was unclear whether this collection was a hematoma or had a microbial origin. Clinical findings, including the mass edema, the redness, and the high temperature of the affected area, led to the decision to perform surgical debridement.

Before the debridement, the patient received preoperative preparation with hydrocortisone, and cortisol levels were monitored and adjusted as necessary during the process. ACTH levels were also closely monitored. The patient underwent another surgical debridement for a new subcutaneous collection, which revealed the presence of *Staphylococcus xylosus* in one of the culture samples. The VAC was removed, and hydrocortisone treatment was modified in response to cortisol level changes.

After the second debridement, the subcutaneous collection gradually improved, and follow-up assessments showed no signs of infection. Eight months later, according to the clinical, laboratory, and imaging findings, no signs of inflammation were present, and the patient successfully resumed his normal daily activities.

It is noteworthy that gabapentin was initially administered as a prophylactic analgesic treatment before tumor excision and megaprosthesis implantation, with doses varying between 300 mg once, twice, or three times a day, depending on the patient’s pain levels at different times, resulting in almost 6 months of administration with breaks inside this period. During the hospitalization for the prosthesis’s revision, the patient received gabapentin at a dosage of 300 mg twice daily. Following the detection of adrenal insufficiency, the gabapentin dosage was reduced to 300 mg once daily and later discontinued after the second adrenal insufficiency diagnosis. During the periods when adrenal insufficiency was detected, the patient was under gabapentin, antibiotic chemoprophylaxis, and LMWH anticoagulant treatment at therapeutic doses.

As for antibiotic chemoprophylaxis, the patient received vancomycin starting from the first pre-operative day at a dosage of 500 mg four times daily intravenously. The same antibiotic scheme was used as chemoprophylaxis before the removal of the spacer and the placement of the new endoprosthesis, along with cefepime at 2 g three times daily for two days. After the first debridement of the second megaprosthesis, vancomycin was administered at an increased dosage of 550 mg four times daily. Following the second debridement, the antibiotic regimen was further escalated to vancomycin at 1 g four times daily, in combination with rifampicin at 600 mg once daily, for 16 days. The patient was discharged and continued to take norfloxacin at a dosage of 400 mg twice daily orally for 14 days as an outpatient. It is important to note that vancomycin levels were consistently maintained at the lowest therapeutic levels when assessed through immunofluorescence at all times the glycopeptide was administered. The levels of vancomycin were estimated according to the hospital’s protocol for vancomycin monitoring every 3 days. The antibiotic regimen was decided after detailed discussion with the pediatric infectious diseases department against an invasive staphylococcal infection in an immunosuppressed patient.

A detailed investigation was conducted on an outpatient basis with CT and MRI, ruling out any other pathology that could be implicated in the studied event. The patient is in good condition and has returned to his daily activities.

**Figure 1:**
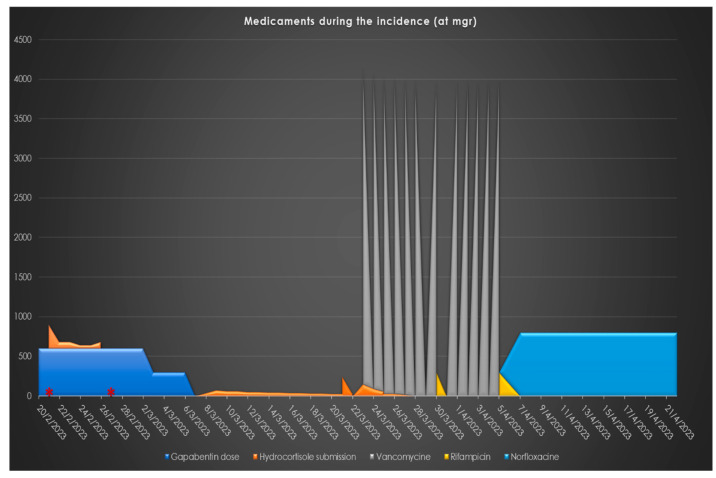
Medicaments related to the case: Their administration (mgrs) through calendar time. (*: Dates when low morning cortisol levels were estimated, indicating HPA axes suppression).

**Figure 2:**
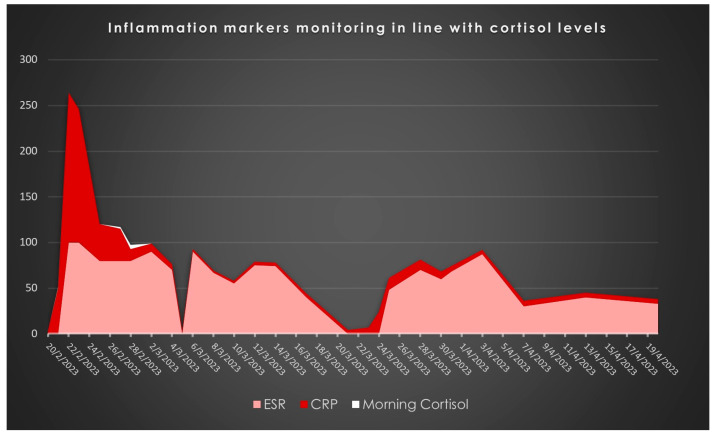
Inflammation-related biomarkers: Their administration (mg/L for CRP and mm ESR) through calendar time.

**Figure 3:**
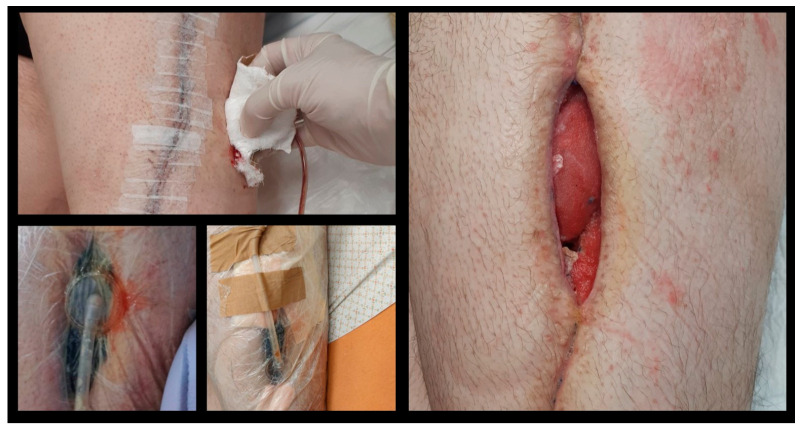
Wound infection clinical image after megaprostesis implantation. *Staphylococcus xylosus* was isolated after debridement’s intraoperative cultures.

Figure 1 represents the administration at mgs regarding the medication related to the presented case, Figure 2 represents the monitoring of infection-related biochemical markers and Figure 3 depicts the clinical images indicating wound infection.

## 3. Review

### 3.1. Gabapentin’s Pharmacological Profile, the HPA Axis and Stress Response

GBP, a GABA analog, is known for its diverse pharmacological effects, which include pain-relieving, anxiety-reducing, and neuroprotective properties. The complex mechanisms of GBP function include its capacity to communicate with brain cells, regulate enzymes such as GAD, and interact with neurotransmitters, including serotonin. The complex pharmacological profile of this substance indicates its possible influence on several physiological systems, including the HPA axis [3,4,17,18,19]. Interestingly, GABAergic synapses’ role in regulating the HPA axis is well-established, and GBP, as a GABA analog, might influence this axis through several mechanisms [3,4,7,8,17,18,19,20,21].

The correlation between GABA and stress is a significant topic in this field. The literature extensively documents the involvement of GABA in regulating the activity of the HPA axis and stress responses. GABAergic synapses have been shown to impact important regulators of the axis, including CRH neurons [4,19,22,23]. Furthermore, the contrast between phasic and tonic inhibition of GABAergic activity, facilitated by distinct GABA receptors, introduces an additional level of intricacy to the regulation of the HPA axis during stressful situations. More precisely, the δ-subunit of GABAARs seems to have a crucial function in regulating neurosteroid responses associated with stress [3,6,19,24].

### 3.2. Gabapentin and Cortisol Levels in Clinical Settings

Several studies provide valuable insights into the complex relationship between GBP and cortisol levels. Karbić et al. (2014) [12] investigated the impact of GBP on plasma cortisol levels and immune status in hysterectomized women, revealing a significant decrease in cortisol levels following GBP administration. In detail, the effects of GBP on cortisol levels were examined in a group of 60 women undergoing abdominal hysterectomy. These patients were randomly assigned to receive either 600 mg of GBP or a placebo one hour before surgery. The results demonstrated that GBP significantly reduced plasma cortisol levels 24 h postoperatively compared to the placebo group. This reduction in cortisol indicates that GBP can suppress the HPA axis’s typical stress response, likely contributing to the observed decrease in postoperative pain [25]. Additionally, GBP was found to reduce catecholamine levels, including epinephrine and norepinephrine, which are also involved in the body’s stress response. This dual effect on both cortisol and catecholamines underscores GBP’s potential role in mitigating surgical stress [26,27]. Further supporting this, Hudec and Griffin (2020) [28] explored the effects of GBP on stress markers in a veterinary context, finding that pre-appointment administration of GBP to cats resulted in a slight but not statistically significant reduction in cortisol levels. This suggests that GBP’s impact on cortisol may be modest and potentially related more to its sedative properties than direct suppression of the HPA axis [28]. However, this effect, though minor in this study, aligns with other findings that indicate GBP may exert a calming effect, reducing stress-induced hormonal responses. Contrasting these findings is a case reported by Alam and Srinivasan [11], where a 56-year-old patient undergoing total knee replacement and receiving GBP as part of postoperative pain management displayed significantly low cortisol levels on the first and fourth postoperative days. Despite these low levels, the patient’s adrenal response to a Short Synacthen Test was normal, suggesting that GBP was likely suppressing the stress response rather than causing primary adrenal insufficiency [7,22,27]. This case highlights the variability in how GBP can affect cortisol levels, particularly under conditions of surgical stress.

Upon comparing these results with the case report presented in this manuscript, we observe a comparable trend in which the injection of GBP was linked to markedly changed cortisol levels, requiring the use of cortisol-replacement treatment. The reported case involves a juvenile patient diagnosed with osteosarcoma who, following the administration of GBP, experienced temporary adrenal insufficiency during a period of heightened surgical stress. These findings are consistent with the existing body of research, indicating that GBP can inhibit the HPA axis’s reaction to stress, which could result in severe decreases in cortisol levels that may need medical intervention. In contrast to the patient described in Alam and Srinivasan’s study [11] who did not experience adrenal insufficiency as a result of cortisol suppression, the patient in this case report did show significant hypocortisolemia that needed therapy. This underscores the need for meticulous surveillance of cortisol levels in patients undergoing GBP therapy, especially in scenarios involving high-stress surgical procedures.

To our knowledge, this is the third clinical case [11,12] underlying that GBP’s effects on cortisol levels are complex and context-dependent. Table 1 summarizes the main key findings of the 3 clinical cases [11,12], involving the one presented in this paper, that have been reported in the literature by now and also the veterinary cohort by Hudec & Griffin [28]. According to the existing literature, GBP can effectively reduce the body’s stress response by lowering cortisol and catecholamine levels, and this effect can vary widely among individuals. Under certain circumstances, such as the one detailed in this paper, GBP might result in substantial adrenal insufficiency, especially during the week before surgery. The results emphasize the requirement of individualized patient care, including the precise monitoring of adrenal function while giving GBP, particularly to patients who are undergoing significant surgeries or have pre-existing susceptibilities to stress reactions [26,27,28].

### 3.3. Clinical Implications of Gabapentin-Induced Adrenal Insufficiency

The potential of GBP to induce adrenal insufficiency, particularly in high-stress settings such as surgery, carries significant clinical implications [29,30]. The ability of GBP to suppress HPA axis and subsequently reduce cortisol levels can be both beneficial and risky, depending on the patient’s overall health status and the context of GBP administration [4,8,12,17].

The case report presented in this manuscript exemplifies the serious consequences that can arise from GBP-induced cortisol suppression. In this case, a young patient with osteosarcoma experienced transient adrenal insufficiency during a high-stress surgical period after prolonged GBP administration. This necessitated adrenal replacement therapy, underscoring the need for careful monitoring and potential intervention when using GBP in similar clinical scenarios.

Supporting this observation, the study by Karbić et al. (2014) [12] provides robust evidence of GBP’s capacity to alter cortisol levels and modulate immune responses in the perioperative setting [13]. In their study involving women undergoing abdominal hysterectomy, GBP significantly reduced plasma cortisol levels 24 h after surgery, compared to a placebo group. This reduction in cortisol was correlated with a decrease in pain scores and catecholamine levels, suggesting that GBP effectively dampens the physiological stress response associated with surgery. However, the study also highlights potential risks, noting that such suppression of cortisol and catecholamines could impair the body’s ability to respond to acute stressors, such as surgery or infection, potentially leading to complications [12,13,18].

Moreover, our findings, along with the findings of the aforementioned studies, introduce the broader implications of GBP-induced immunomodulation [3,7,12,13]. By suppressing cortisol and catecholamine levels, GBP can influence immune function, as these hormones play a crucial role in regulating inflammation and immune responses [26,27]. The study found that GBP altered the proportions of various lymphocyte subsets, potentially impacting the body’s ability to mount an effective immune response postoperatively [12,26,27,31]. This is particularly relevant in surgical patients, who are at increased risk for infections and other complications when immune function is compromised.

The clinical implications of these findings are clear: healthcare providers must weigh the benefits of GBP in controlling pain and reducing stress responses against the potential for inducing adrenal insufficiency and compromising immune function. This is especially critical in patients undergoing major surgeries or those with pre-existing conditions that may predispose them to adrenal insufficiency. Regular monitoring of cortisol levels and immune markers in such patients is advisable to detect and address any adverse effects promptly. While GBP is a valuable tool in perioperative pain management, its impact on the HPA axis and immune function necessitates a cautious approach, particularly in high-risk patients. Further research is needed to develop guidelines that balance the analgesic and stress-reducing benefits of GBP with the need to maintain adequate adrenal and immune function, ensuring patient safety and optimal outcomes in the surgical setting.

### 3.4. Gabapentin-Induced Hypocortisolemia and Infection Risk

The connection between gabapentin-induced hypocortisolemia and subsequent infection in this case report cannot be overlooked. The initial administration of GBP raised questions about its role in modulating the patient’s stress response, as it led to a profound reduction in cortisol levels. The subsequent infection with *Staphylococcus hominis*, which occurred two months after megaprosthesis implantation, may have been facilitated by this compromised stress response, as cortisol is a crucial component of the body’s immune defenses. Hypocortisolemia resulting from GBP might have contributed to the patient’s increased susceptibility to infection [31]. It is well known that glucocorticoids affect both the innate and adaptive immune systems through a variety of mechanisms [32]. Thus, the reduction in these adaptations, in combination with the stress provoked by the surgical procedure and the fact that the natural barrier against pathogens had been injured due to the extensive surgical incision, put the patient at a disadvantage against potential invaders and led to infection [6,26,31,32].

This case vividly illustrates how the complex interplay between GBP and cortisol can have substantial clinical implications, particularly in the context of major surgical procedures, as it may affect a patient’s ability to mount an effective immune response against potential pathogens. The emergence of *Staphylococcus xylosus* in this case further underscores the intriguing connection between gabapentin-induced hypocortisolemia and the development of infections [33]. The patient’s altered adrenal response due to gabapentin likely contributed to a weakened immune defense, increasing susceptibility to infections, including unusual pathogens like *Staphylococcus xylosus* [33,34]. Complications included infections with *Staphylococcus hominis* and *Staphylococcus xylosus*, pathogens typically considered low virulence but capable of causing significant infections in immunocompromised hosts [35,36]. The presence of *Staphylococcus xylosus* is particularly noteworthy, as it is a coagulase-negative staphylococcus (CoNS) and is generally considered a contaminant [35,36]. However, in this immunosuppressed state induced by GBP, *Staphylococcus xylosus* emerged as a primary pathogen, underscoring the importance of considering the patient’s immunological status when interpreting microbiological findings [33,34,35,36].

This scenario aligns with findings from the study by Brand and Rufer (2021) [33], where *Staphylococcus xylosus* was identified as the causative agent in a late prosthetic joint infection (PJI). In this study, a 70-year-old male patient developed a knee joint infection 18 years after total knee arthroplasty (TKA), which was attributed to *Staphylococcus xylosus* [33]. The patient’s history included recent trauma and possible sources of hematogenous seeding, which likely contributed to the infection [33]. The study emphasized that CoNS, including *Staphylococcus xylosus*, should not be routinely dismissed as contaminants, particularly in immunocompromised or stressed patients, where these organisms can act as opportunistic pathogens [33].

Similarly, the study by Scorzolini et al. (2014) [34] demonstrated the increased sensitivity of microbiological detection methods such as sonication in identifying pathogens like CoNS in prosthetic joint infections (PJIs) [34]. This study highlighted the importance of accurately diagnosing and treating PJIs, particularly in patients with compromised immune systems, where pathogens like *Staphylococcus xylosus* can cause significant infections despite their typically low virulence [33,34,35,36]. The study’s findings further support the need for vigilance in interpreting culture results in the context of GBP-induced immunosuppression.

When comparing the patients in the two previous reported studies to the patient in our case report, several similarities emerge. All patients were immunocompromised, either due to their underlying conditions, surgical stress, or GBP-induced hypocortisolemia, which facilitated the emergence of *Staphylococcus xylosus* as a significant pathogen. In both the Brand and Rufer case and our case report, *Staphylococcus xylosus* caused serious infections that required intensive treatment [33]. These cases emphasize the clinical significance of GBP-induced cortisol suppression, which can impair the body’s ability to fend off infections, especially from opportunistic pathogens like CoNS. The presence of *Staphylococcus xylosus* as the main pathogen in these cases underscores the need for careful consideration of the potential for GBP to exacerbate infection risks in vulnerable patients [33,34,35].

**Figure 4:**
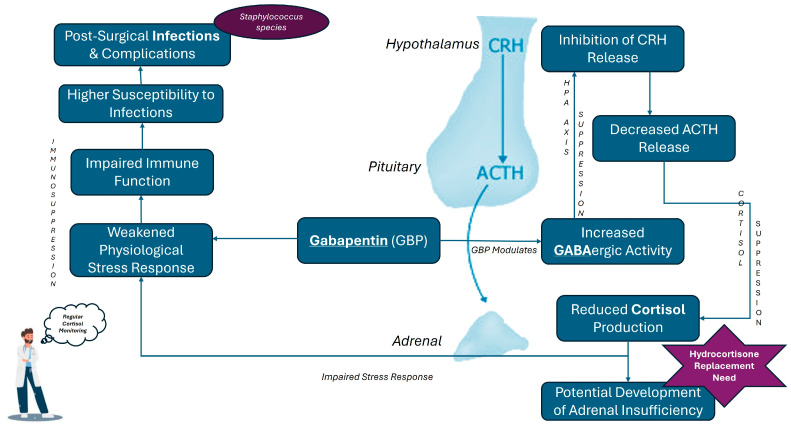
Mechanistic Pathways of Gabapentin-Induced Cortisol Suppression and Related Disease Risks.

To sum it up, the risk of infection in patients receiving GBP, particularly those undergoing surgery, is heightened due to the drug’s impact on cortisol levels and immune function. The emergence of *Staphylococcus xylosus* as a significant pathogen in these cases serves as a reminder that even typically low-virulence organisms can cause severe infections in the context of GBP-induced immunosuppression. Clinicians should be aware of these risks and consider them when managing patients on GBP, especially in perioperative settings or in those with pre-existing conditions that may compromise their immune responses. Figure 4 summarizes the mechanisms through which GBP may alter the natural stress response and lead to infection susceptibility via HPA axis suppression.

## 4. Conclusions

- Complex Interplay: The review and case report highlight the intricate relationship between GBP use, adrenal insufficiency, and increased infection risk, particularly in patients undergoing major surgeries. It is also highlighting the principles of adverse effects management as the patients were not monitored for HPA axis and the hypocortisolemia founded during basic routine pre- or post-operative tests. It emphasizes the need for always careful and deep clinical brainstorming when patients with a controversial history suddenly present irregularities in vital axes, such as the glucocorticoids’ axis.
- HPA Axis and Cortisol Regulation: The findings underscore the significance of understanding GBP’s diverse effects on the hypothalamic–pituitary–adrenal (HPA) axis and cortisol regulation.
- Need for Vigilant Monitoring: There is a crucial need to carefully monitor patients receiving GBP, especially in perioperative settings, to mitigate potential risks.
- Future Research: Further research is necessary to clarify the mechanisms behind GBP’s impact on the HPA axis and to develop evidence-based guidelines for managing these cases.

## Figures and Tables

**Table 1:** Summary of Clinical Cases Examining the Relationship Between Gabapentin (GBP) Administration and Cortisol Levels.

Study/Case Report	Patient Characteristics	Medical Condition	GBP Dosage	Effect on Cortisol Levels	Other Findings
Karbić et al. (2014) [12]	60 women, undergoing hysterectomy	Abdominal Hysterectomy	600 mg, 1 h pre-surgery	Significant reduction 24 h post-op	Reduced catecholamines (epinephrine, norepinephrine), mitigated surgical stress
Hudec and Griffin (2020) [28]	Veterinary context (cats)	Pre-appointment stress	25.0–30.5 mg/kg	Slight, non-significant reduction	Suggests effects may be due to sedative properties rather than direct HPA axis suppression
Alam and Srinivasan (2017) [11]	56-year-old male, post-knee replacement	Total Knee Replacement	100 mg 3 times per day	Significantly low on days 1 and 4 post-op	Normal adrenal response to Short Synacthen Test, indicating stress response suppression without adrenal insufficiency
Current Case (2024)	17-year-old boy with osteosarcoma	Megaprostheses placement revision surgeries	300 mg twice daily before revision surgery, tapered to 300 mg once daily before discontinuation	Morning cortisol levels dropped to 1.18 μg/dL, below normal range, necessitating hydrocortisone replacement therapy	Developed transient adrenal insufficiency, requiring multiple interventions; GBP administration associated with suppressed cortisol levels and complex clinical management

## Data Availability

No new data were created or analyzed in this study. Data sharing is not applicable to this article.
